# Comparison of a Resonant Mirror Biosensor (IAsys) and a Quartz Crystal Microbalance (QCM) for the Study on Interaction between *Paeoniae Radix* 801 and Endothelin-1

**DOI:** 10.3390/s8128275

**Published:** 2008-12-15

**Authors:** Jiadong Huang, Qing Lin, Jinghua Yu, Shenguang Ge, Jing Li, Min Yu, Zixia Zhao, Xinsheng Wang, Xiuming Zhang, Xiaorui He, Liang Yuan, Huijun Yin, Tetsuo Osa, Keji Chen, Qiang Chen

**Affiliations:** 1 College of Medicine and Life Sciences, University of Jinan, Jinan 250022, P.R. China; E-Mails: chm_huangjd@ujn.edu.cn (Jiadong Huang); yl860131@126.com (Liang Yuan); 2 College of Chemistry and Chemical Engineering, University of Jinan, Jinan 250022, P.R. China; E- Mails: linqing19851215@126.com (Qing Lin); yujinghua@vip.sina.com (Jinghua Yu); chm_gesg@ujn.edu.cn (Shenguang Ge); 19840321xm@163.com (Xiuming Zhang); hxr666666@126.com (Xiaorui He); 3 Medical College of Nankai University, Tianjin 300071, P.R. China; E-Mail: stellarli@nankai.edu.cn (Jing Li); 4 College of Life Science, Nankai University, Tianjin 300071, P.R. China; E-Mails: zhaozixia@hotmail.com (Zixia Zhao): wangxsh2001@163.com (Xinsheng Wang); flyfish113@163.com (Min Yu); 5 Xiyuan Hospital, China Academy of Traditional Chinese Medicine, Beijing 100091, P.R. China; E- Mails: huijunyin@yahoo.com.cn (Huijun Yin); kjchen@public.bta.net.cn (Keji Chen); # Emeritus professor of Graduate School of Pharmaceutical Sciences, Tohoku University, Aramaki, Aoba-ku, Sendai 980-8578, Japan; E-Mail: osa@mars.dti.ne.jp (Tetsuo Osa)

**Keywords:** Resonant mirror biosensor, IAsys, Quartz crystal microbalance, Endothelin-1 (ET-1), *Paeoniae Radix* 801, Interaction

## Abstract

A resonant mirror biosensor, IAsys, and a quartz crystal microbalance (QCM) are known independently as surface sensitive analytical devices capable of label-free and *in situ* bioassays. In this study, an IAsys and a QCM are employed for a new study on the action mechanism of *Paeoniae Radix* 801 (*P. radix* 801) by detecting the specific interaction between *P. radix* 801 and endothelin-1 (ET-1). In the experiments, ET-1 was immobilized on the surfaces of the IAsys cuvette and the QCM substrate by surface modification techniques, and then *P. radix* 801 solution was contacted to the cuvette and the substrate, separately. Then, the binding and interaction process between *P. radix* 801 and ET-1 was monitored by IAsys and QCM, respectively. The experimental results showed that *P. radix* 801 binds ET-1 specifically. The IAsys and QCM response curves to the ET-1 immobilization and *P. radix* 801 binding are similar in reaction process, but different in binding profiles, reflecting different resonation principles. Although both IAsys and QCM could detect the interaction of *P. radix* 801 and ET-1 with high reproducibility and reliability through optimization of the ET-1 coating, the reproducibility and reliability obtained by IAsys are better than those obtained by QCM, since the QCM frequency is more sensitive to temperature fluctuations, atmospheric changes and mechanical disturbances. However, IAsys and QCM are generally potent and reliable tools to study the interaction of *P. radix* 801 and ET-1, and can conclusively be applied to the action mechanism of *P. radix* 801.

## 1. Introduction

Resonant mirror biosensor (IAsys) and quartz crystal microbalance (QCM) have been known independently as surface sensitive analytical devices capable of on-line monitoring of interfacial reactions and thin film depositions [1-3]. IAsys is based on an optical measurement technique that probes the thickness and dielectric constant of thin films at a cuvette surface [4-8]. QCM measures the changes in acoustic thickness or mechanical resonance properties of a thin film deposited on a metal electrode (e.g. gold, silver, and copper, etc.) [9-11]. As surface analytical tools, each of IAsys and QCM has its own specific features, weaknesses, and assumptions inherent in data collection and analysis. Combined IAsys and QCM data collection and analysis allow one to take the advantage of the features, to test the validity of the assumptions and to gain a better understanding of a specific interfacial reaction. Both the devices have been widely used for biological analysis, clinical diagnosis, and environmental monitoring [12-13]. The liquid cell configuration of the two devices makes them suitable for real-time study of bioaffinity reactions in relevant solution conditions of temperature, flow rate, pH and ionic strength [14-15]. In the recent years, the applications of IAsys and QCM for biological analyses have been reported increasingly, including immunoassay assay of BSA [16], enterotoxin detection [17], enzymatic analysis for hydrogen peroxide quantification [18], and blood plasma coagulation determination [19].

*Paeoniae Radix* 801 (*P. radix* 801), a component of *Radix Paeonia Rubra*, is mainly composed of propyl gallate. It is one of the active ingredients of the blood-activating and stasis-eliminating traditional Chinese medicine (TCM) [20]. By inhibiting the formation of the blood platelet Thromboxane A_2_ (TXA_2_), *P. radix* 801 has many pharmacological functions, such as accelerating the formation of artery inner prostaglandin (PGI2) [21], inhibiting the formation of thrombus, resisting blood platelet aggregation [22], etc. It has been applied to cure coronary heart disease, angina, etc. [23, 24]. However, there is no detailed research so far on the action mechanism and dynamics of *P. radix* 801, limiting its further applications. The cytokine endothelin-1 (ET-1) is a kind of polypeptide composed of 21 amino acids which is produced and released by endothelial cells. When endothelial cells are damaged or their functions become maladjusted, the secretion of ET-1 will increase. Its molecular weight is estimated to be 2,492.0 Da [25]. ET-1 is not only one of the strongest vasoconstrictors but also is evaluated as a platelet aggregation promoting factor, having become the most popular target of various blood-activating and stasis-eliminating TCM [26-28].

In the present paper, a cuvette-based IAsys and a 9 MHz QCM were employed to study the specific binding and interaction process between *P. Radix* 801 and ET-1 in real time in order to find out the mechanism of *P. radix* 801. The ET-1 coating mass was optimized. Comparison of the two devices with respect to reproducibility and reliability are conducted.

## 2. Results and Discussion

### 2.1. ET-1 Immobilization on the surface of IAsys cuvettes

**Figure 1.**
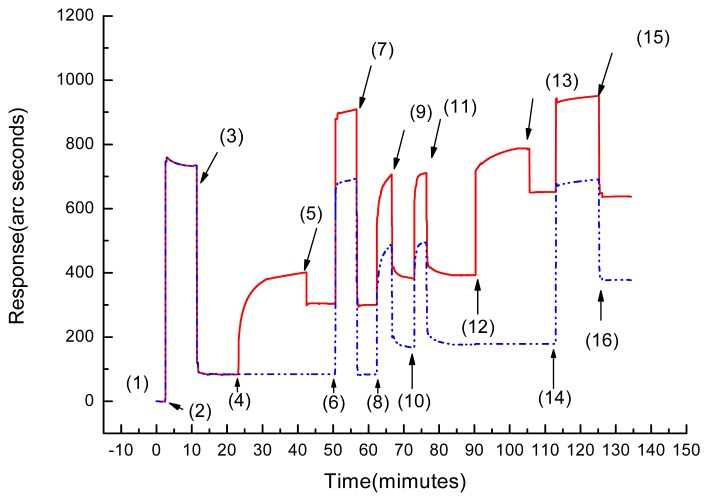
Resonance response of IAsys to the ET-1 immobilized CMD cuvette and binding detection of *P. radix* 801(pH 7.4 PBST, 0.18 mg mL^-1^ ET-1, 5 mg mL^-1^ *P. radix* 801)

As mentioned in Experimental Section, the ET-1 molecules were immobilized on the surface of the IAsys cuvette by the ester exchange reaction of NHS to the COOH groups of CMD. Figure1 shows the response of IAsys to ET-1 immobilization and the response of *P. radix* 801 to ET-1 bound on the immobilized IAsys.

In Figure 1, (1) corresponds to the response of a baseline of IAsys cuvette stabilizated by buffer; (2) is the response by addition of the EDC/NHS mixture into the cuvette; (3) is the response by buffer washing to remove the unreacted EDC/NHS mix0ture; (4) is the response by addition of ET-1 (0.18 mg mL^-1^) and led to a typical irreversible response, representing a time-dependent accumulation of the macromolecules on the surface; (5) is the response by buffer washing to remove unreacted ET-1; unreacted ET-1 was removed and the response decreased to point (6). The increase in refractive index by immobilization of ET-1 was given by the subtraction of the baseline levels of point (6) – point (4). The binding amount of ET-1 was calculated according to the relation that 1 ng mm^-2^ molecules correspond to a shift of 200 refractive arc seconds described in section *2.2*. Therefore, the binding amount of ET-1 was obtained as 1.093 ng mm^-2^ by the shift of refractive index of 218.62 arc seconds.

**Figure 2.**
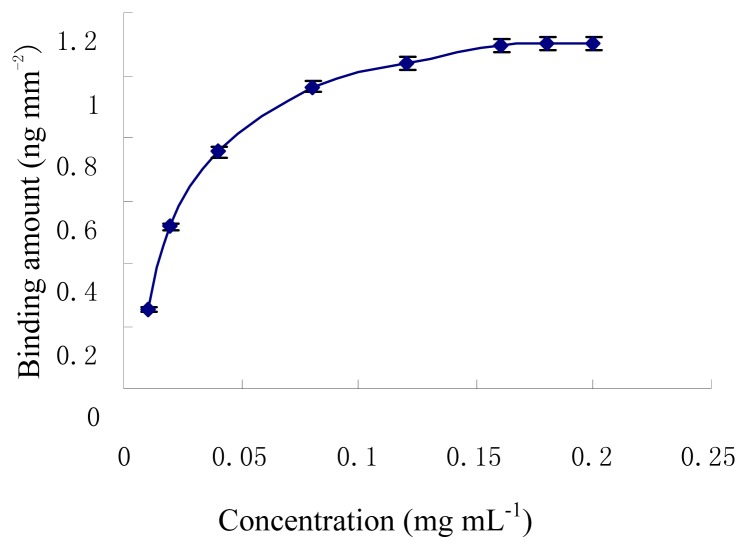
Optimization of ET-1 immobilization on the IAsys cuvette (pH7.4 PBST)

The immobilization of different concentrations of ET-1 on the cuvettte was studied. The ET-1 concentrations were varied from 0.01 to 0.20 mg mL^-1^. Figure 2 shows the effect of ET-1 concentration on the response of the IAsys. The biosensor response increased from 0.01 to 0.18 mg mL^-1^ and then almost saturated to keep constant. Hence in all subsequent IAsys experiments, the concentration of ET-1 was maintained at 0.18 mg mL^-1^.

### 2.2. Detection of the binding between P. radix 801 and ET-1 by IAsys

Point (6) in Figure 1 marks the response by blocking of the non-coupled activated CMD sites with ethanolamine; (7) is the response by buffer washing to remove ethanolamine; (8) is the response by removing unbound ligand molecules with formic acid; (9) is the response by buffer washing to remove formic acid; point (10) – point (11) are the responses of repeating (8) - (9); (12) is the response by addition of *P. radix* 801; the shift in refractive index and thickness at the sensing surface occurred upon binding of *P. radix* 801 to ET-1, increasing the response with an irreversible curve. The reaction was continued until reaching an equilibrium state. Then PBST was added to wash out free *P. radix* 801 (13) and the response decreased to point (14). The increase in refractive index by adsorption of *P. radix* 801 was given by the subtraction of point (14) – point (12) and obtained as 259.40 arc seconds. Therefore, the binding amount of *P. radix* 801 coupled onto the surface could be calculated as 1.297 ng mm^-2^ as described in Section *2.2*. (14) is the response by treating with formic acid (12 min of reaction) to remove *P. radix* 801 to regenerate the immobilized ET-1. (15) is the response by PBST washing to remove formic acid, and the response decreased to baseline (16) whose value was same as that of baseline (12). It shows that *P. radix* 801 is effectively removed from immobilized ET-1 and the ET-1 bound cuvette can be used again for measurement.

A reference or blank channel without anchoring ET-1 gave no binding signal (broken line in Fig.1). It shows that *P. radix* 801 was removed with easy washing on the unmodified cuvette surface and there is no specific binding or non-specific adsorption of *P. radix* 801. This also indicates that *P. radix* 801 bound ET-1 on the cuvette surface specifically.

### 2.3. ET-1 immobilization on the gold substrates of QCM

**Figure 3.**
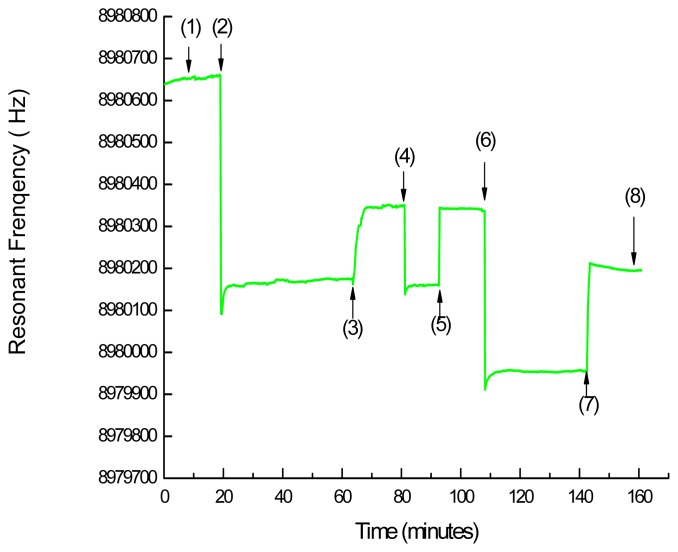
QCM sensorgram by the immobilization amount of ET-1 (2)–(4) and the adsorption amount of *P. radix* 801 (6)–(8) (pH 7.4 PBST, 1.8 mg mL^-1^ ET-1, 10 mg mL^-1^ *P. radix* 80*1)*.

Figure 3 shows the response of a QCM to the ET-1 immobilizing gold substrate with *P. radix* 801. Response (1)–(2) is at the equilibration and a stabilized baseline of the EDC/NHS treated SAM surface with α-TA after the addition of PBST. Response (2) corresponds to the addition of ET-1. The resonance frequency of QCM was simultaneously dropped. The curve abruptly decreases and reaches (3). The gold substrate was rinsed with deionized water to remove unreacted ET-1, the curve reaches to (4). (2)–(4) showed that ET-1 was immobilized on the surface of substrate. The adsorbed value of ET-1 on the surface of substrate was obtained as 2.774 ng mm^-2^ by the equation (1). Response (4)–(5) is the process to protect non-coupled activated NHS of SAM with ethanolamine. Response (5) is buffer washing to remove excess ethanolamine.

**Figure 4.**
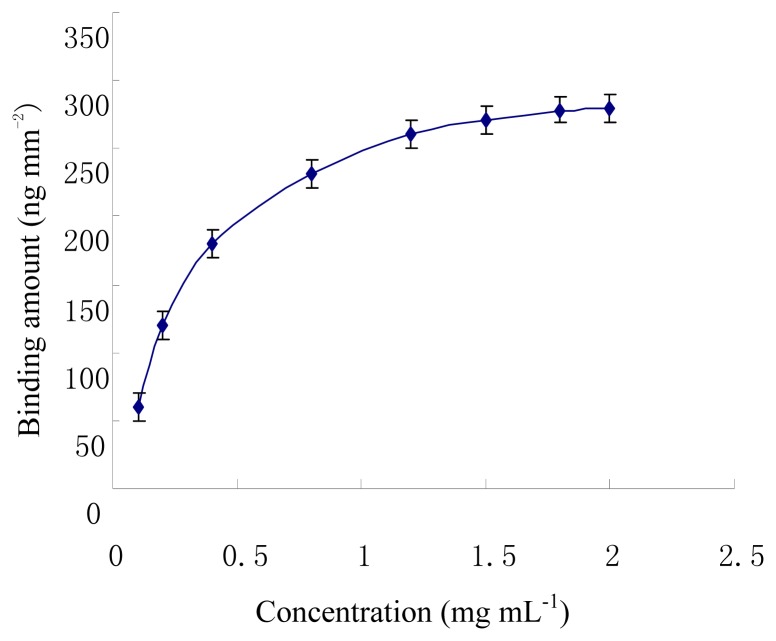
Optimization of ET-1 concentration to immobilize on the QCM substrate(pH 7.4 PBST)

The optimum concentration of ET-1 immobilized on the substrate was studied. The concentrations of ET-1 solution were varied from 0.1 to 2.0 mg mL^-1^. Figure 4 shows the effect of the ET-1 concentration on the response of the QCM. The response increased from 0.1 to 1.8 mg mL^-1^, and then almost saturated to keeping constant. Hence, in all the subsequent QCM experiments, the concentration of ET-1 was maintained at 1.8 mg mL^-1^.

### 2.4. Detection of the binding between P. radix 801 and ET-1 by QCM

The subsequent addition of *P. radix* 801 gave a typical irreversible response (6) in Figure 3. Response (7) is buffer washing to remove unreacted *P. radix* 801 and the curve reached (8). Therefore, the subtraction value (6)–(8) corresponds to a frequency increase (Δ*F)* by the adsorption of *P. radix* 801, indicating a rapid accumulation of the *P. radix* 801 molecules on the surface. The adsorbed values of *P. radix* 801 on the substrate were calculated as 1.303 ng mm^-2^. The results mentioned above are concluded that *P. radix* 801 binds ET-1.

The specific binding between *P. radix* 801 and ET-1 was evaluated by a blank control experiment without anchoring ET-1 on the substrate surface. No detectable binding signal was obtained, showing that *P. radix* 801 binds ET-1 specifically on the substrate surface. The detail interaction, however, between the main component of *P. radix* 801, propyl gallate of a strong anti-oxidant, and a polypeptide of ET-1 composed of 21 amino acids is not clear.

### 2.5. Reproducibility and reliability of the IAsys and QCM applied to detect the binding between P. radix 801 and ET-1

The same binding experiments between *P. radix* 801 and ET-1 as described above were repeated 4 times further, respectively, in order to investigate the reproducibility and reliability of these two kinds of affinity biosensing methods. The results were presented in Tables 1 and 2. Both results show that IAsys and QCM exhibit sufficient reproducibility and reliability. So it can be concluded that these affinity methods have high reproducibility and reliability in exploring the binding and the interaction between *P. radix* 801 and ET-1.

However, the reproducibility of QCM data is slightly lower than that of the IAsys. Since both biosensing methods adopt a similar liquid-handling method, the lower reproducibility of the QCM might be derived from more environmental sensitivity, i.e. frequency drifts due to temperature fluctuations, atmosphere changes and mechanical disturbances.

### 2.6. Comparison of IAsys and OCM measurements to ET-1 immobilization and the interaction between ET-1 and P. radix 801

In the present study, two kinds of affinity biosensors of IAsys and QCM were applied to monitoring the interaction between *P. radix* 801 and ET-1. The sensorgrams of IAsys and QCM measurements were compared, the response time of QCM was faster than that of IAsys. Compare (2) – (3) in Figure 3 with (4) – (5) in Figure 1 for ET-1 addition and (6) – (7) in Figure 3 with (12) – (13) in Figure 1 for interaction with *P. radix* 801. The response curve for QCM was more quickly saturated and could make on-line measurement short. On the other hand, the curve for IAsys tended to have a typical irreversible behavior. An assumption may be that the immobilization amounts of α-TA on QCM surface was so much that provided much more carboxylic groups than that on IAsys surface. Furthermore, the more carboxylic groups bound more ET-1 on QCM surface. So the response curve for QCM was more quickly saturated.

The immobilization methods of ET-1 are different in both methods. The preliminary results showed that the average immobilized amount of ET-1 in IAsys is 1.097 ng mm^-2^, on the other hand, that in QCM is 278.880 ng cm^-2^ (2.79 ng mm^-2^), in spite of the almost same interaction amounts of P. radix 801 in both measurements. It might depend on the difference of the surface states. The more carboxylic groups on QCM surface bound more ET-1. From the depicts in this and last paragraphs, it can be concluded that the present immobilization method of ET-1 on QCM surface was effective and could be controlled without difficulty.

P. radix 801 bound ET-1 specifically and its average binding masses on the cuvette and gold substrate were 1.301 ng mm^-2^ and 131.080 ng cm^-2^ (=1.31 ng mm^-2^), respectively. The IAsys and QCM response curves to the ET-1 immobilization and P. radix 801 binding show similar trends but different in shape. This may arise from the different sensing principles.

Based on the experimental results, it can be said that IAsys and QCM, each has its own advantages and disadvantages (limitations) when used in the study on the interaction between *P. radix* 801 and ET-1. IAsys has better reproducibility and reliability, relatively simple, but is more expensive. QCM setup needs relatively complicate instruments, and less expensive, but the frequency measurement is more environmentally sensitive. It is well-known that liquid phase QCM responses are attributed to several factors, including mass uptake, density and viscosity of liquid samples, and viscoelasticity of the molecular coating [42]. Water bound or hydro-dynamically coupled to the protein layer will also result in additional frequency shifts. Table 3 summarizes the features of IAsys and QCM techniques when used for studies on the molecular interaction.

## 3. Experimental Section

### 3.1. Reagents

1-Ethyl-*N*-(3-dimethylaminopropyl)carbodiimide hydrochloride (EDC), *N*-hydroxysuccinimide (NHS), α-thioctic acid (α-TA), and ethanolamine were obtained from Sigma-Aldrich (St. Louis, MO, USA). Endothelin-1 (ET-1) was purchased from Alexis (Lausen, Switzerland). Phosphate buffered saline/Tween-20 (PBST, pH 7.4) was composed of 0.01 M Na_2_HPO_4_/NaH_2_PO_4_, 0.138 M NaCl, 0.0027 M KCl and 0.05% Tween-20. *Paeoniae Radix* 801 for injection was purchased from Fu Jian Lijiexun Medicine Co., Ltd (Fuzhou, China). The carboxymethyldextran (CMD) dual-well cuvette was purchased from Labsystems Affinity Sensors (Cambridge, UK). All solutions were made by using deionized water. All reagents were of analytical grade and were used without further purification.

### 3.2. IAsys device

The IAsys series of optical biosensors (Labsystems Affinity Sensors, Cambridge, UK) are sophisticated analytical instruments schematically depicted in the literature [29-32] utilizing advanced resonant mirror (RM) optical biosensor technology. The binding of molecules to the chip produces a change in the refractive index on the biosensor surface. Since the refractive changes are proportional to the changes in the adsorbed mass, this technique allows the quantitative analysis of interacting with the ligand immobilized on the sensor chip. The biosensor consists of a cuvette (pre-derivatized with CMD, 1 to 80 µL reagent capacity, 4 mm^2^ bottom area.). Reagents are injected into the cuvette for the formation of binding. The instrument has been employed to investigate and quantify subtle differences in k_A_ (Association equilibrium constant or Affinity constant) and k_D_ (Dissociation equilibrium constant) while revealing characteristics and functions of biomolecules resulting from the specific interaction of a variety of binding partner systems. In addition, the instrument is also applied to the study on molecular recognition, determination of ligand concentration and multi-molecular interactions. The IAsys can accomplish a measurement purpose quickly and in real time, and eliminate the usage of radiolabels or other chemical tags. Compared to the traditional methods, numerously necessitating additional steps are saved [33-34]. As molecules with a refractive index different from the bulk solution bind to the sensor surface, the critical resonant angle is changed. This shift generates a signal and is expressed in arc seconds. Based on the guidelines supplied by the manufacturer [35], the sensitivity of the biotin cuvette is 600 arc seconds per ng/mm^2^. That is, 200 arc seconds of resonance angle change corresponds to immobilization of approximately 1 ng protein molecules onto 1 mm^2^ surface. This number may be used to calculate the density and the amounts of immobilized protein.

### 3.3. QCM device

The QCM system employed in this study consists of an AT-cut 9 MHz quartz crystal purchased from *SEIKO EG&G*. Five V dc was applied to the circuit to drive the crystal substrate and the frequency was monitored with a frequency counter (Model QCA917, *SEIKO EG&G)* connected to a computer. The quartz crystal was employed as a working electrode. The application of a small change in mass Δ*m* on the surface of the crystal results in a proportional shift in resonant frequency Δ*F*. This relationship was firstly described by Sauerbrey [36] with the equation (1):

(1) $$\Delta F=-2{F_{0}}^{2}\Delta m/A{\left(\rho_{q}\mu_{q}\right)}^{1/2}$$

where *F_0_* is the fundamental oscillation frequency of the dry crystal, *ρ_q_* is the density of quartz (2.65 g/cm^3^) and *µ_q_* is the shear modulus (2.95×10^11^ dyne cm^-2^), A is the electrode area (0.20 cm^2^). For the 9 MHz quartz crystals used in this work, equation (1) predicts that a frequency change of 1 Hz corresponds to a mass increase of 0.89 ng mm^-2^. It is a kind of transducer capable of sub-nanogram mass measurement *in vacuo* [36] and gases [37]. They have been shown to be capable of the measurement of changes in solution properties [38].

### 3.4. IAsys Experiment

The IAsys was operated at 25 °C. The time interval between data collection taken by IAsys was 1.0 s. The percentage value specifying the amplitude of stirrer oscillation at 140 Hz was 100% in order to minimize mass transport effects.

#### 3.4.1. Immobilization of ET-1 on the Surface of the Cuvette

**Scheme 1.**
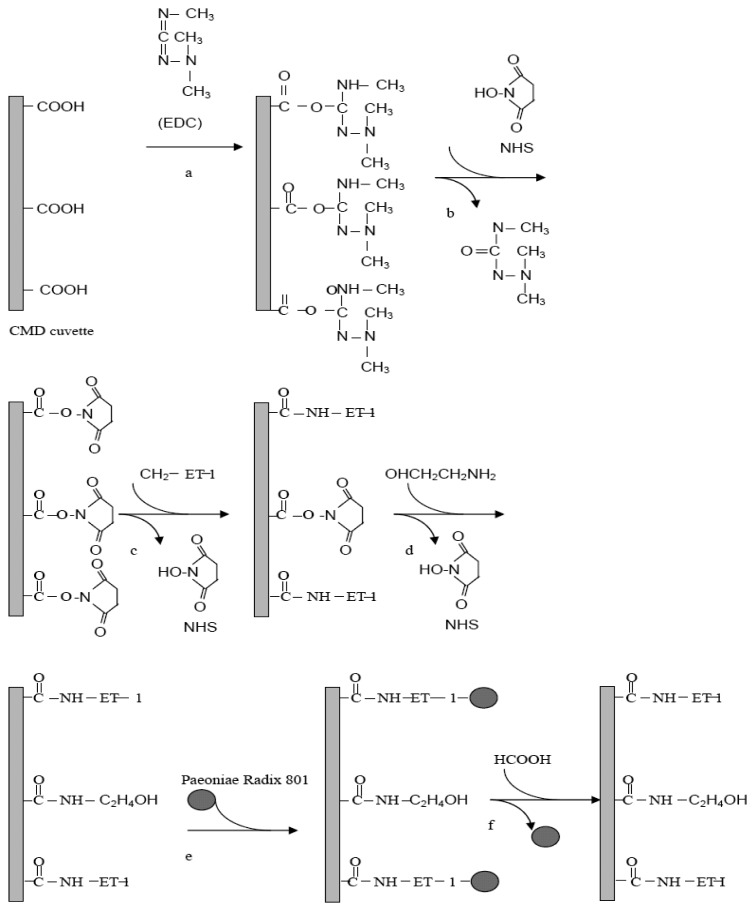
Immobilization of ET-1 on CMD cuvette via activation with EDC/NHS and binding of *P. radix* 801.

The CMD hydrogel in the dual cell was activated with a 1:1 EDC/NHS mixture (10 µL of 100 mg mL^-1^ EDC and 10 µL of 100 mg mL^-1^ NHS) for 9 min. The function of the EDC/NHS is to activate the surface and promote the formation of the covalent linkages by forming the *N*-hydroxysucciniimide ester (Scheme 1a and b). PBST was added to remove the unreacted EDC/NHS mixture and to stabilize the baseline. Then, the different concentrations of ET-1 (5 µL) were added into the NHS activated cuvette and incubated for 20 min to equilibrate. The active NHS intermediates under the action of EDC were replaced by the primary amines of protein (ET-1) to make the amido bond on the surface of CMD (Scheme 1c) [39,40]. ET-1 was immobilized only in one channel of the cuvette. The second channel was used as a reference channel. The running buffer was PBST. The optimum concentration of ET-1 is discussed later.

#### 3.4.2. Detection of the Binding between ET-1 and P. radix 801

an ethanolamine solution (40 μL) was added into the cuvette (Scheme 1d) to block the non-coupled activated CMD sites (reaction: 5 min). Then, excess ethanolamine was removed by buffer washing. A 1 M formic acid solution (40 μL) was added into the cuvette to remove the unbound ligand (ET-1) molecules (reaction: 5 min). Then buffer washing was carried out to remove formic acid. This process was repeated two times. PBST re-equilibration and baseline stabilization were performed. (reaction: 15 min) by washing 3 times with PBST. Then, a *P. radix* 801 (25 μL, 10 mg in 1 mL PBST) solution was added into the cuvette containing PBST (25 μL, therefore the final concentration of *P. radix* 801 was 5 mg mL^-1^) and the cuvette was incubated for 16 min to equilibrate. The refractive index was recorded. The amount of the immobilized *P. radix* 801 was determined by the method described above. The progressed interaction scheme of *P. radix* 801 on the ET-1-bound cuvette surface is schematically shown in Scheme 1e. Finally, formic acid was added into the cuvette to remove *P. radix* 801 to regenerate the immobilized ET-1(Scheme 1f).

### 3.5. QCM Experiment

#### 3.5.1. Immobilization of ET-1 on the Surface of the QCM Gold Substrates

The gold substrate surface was cleaned by placing a drop of Piranha solution (3:1H_2_SO_4_/30%H_2_O_2_) [41]. *CAUTION: Piranha solution should be handled with extreme care and only small volumes should be prepared at any one time*. Piranha solution was spread on the gold surface for 3 min. Then, the substrate was rinsed thoroughly with deionized water. This process was repeated three more times.

**Scheme 2.**
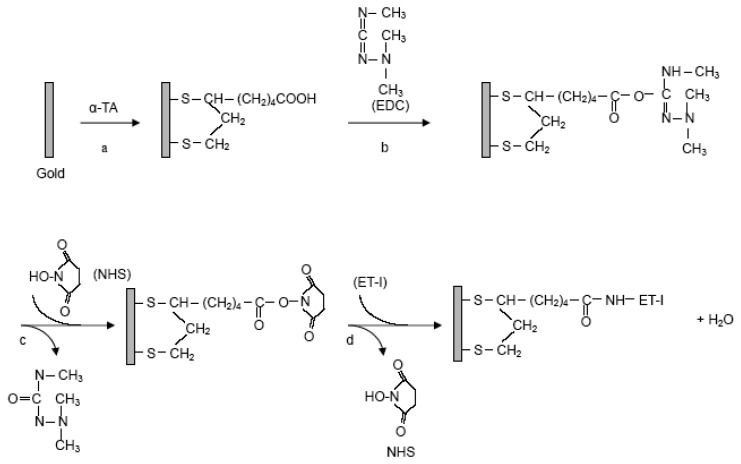
Immobilization of ET-1 on α-TA treated gold substrate via activation with EDC/NHS.

Furthermore, the gold substrate was cleaned by using acetone for 30 min and incubated in anhydrous methanol for 30 min. By the addition of 5 mM α-TA solution (20 μL) on the surface of the substrate for 50 min. Disulfides are known as a kind of molecules to form a stable self-assembled monolayer (SAM) on a gold surface owing to the strong S-Au covalent bond [41]. Then the SAM of α-TA was formed on the gold substrate (Scheme 2a). After the treated substrate was rinsed with deionized water for 3 times, a mixture of 100 mg mL^-1^ EDC (10 µL) and 100 mg mL^-1^ NHS (10 µL) was dropped to the SAM of α-TA on the gold surface. The function of the EDC/NHS is to activate the surface and promote the formation of the covalent linkages by forming the *N*-hydroxysucciniimide ester (Scheme. 2b and c).

Then different concentrations of ET-1 (10 µL) were added onto the NHS activated gold surface for 50 min at room temperature. The active NHS intermediate under the action of EDC is generally replaced by the primary amines of protein to make the amido bonds. Thus, ET-1 was covalently immobilized on the gold substrate by the amido bond (Scheme 2d). The optimum concentration of ET-1 is discussed later. Then the substrate was rinsed thoroughly with deionized water.

#### 3.5.2 Detection of the Binding between ET-1 and P. radix 801

Unreacted NHS-esters were protected with 1 M ethanolamine, pH 8.5. Ten μL of *P. radix* 801 solution (10 mg in 1 mL PBST) was added onto the surface of the gold substrate modified by ET-1 to equilibrating for 20 min. The frequency response was recorded. The amount of the immobilized *P. radix* 801 was determined by the equation (1).

## 4. Conclusions

IAsys and QCM measurements have high reproducibility and reliability and can be employed as reliable tools for studies on the interaction of *P. radix* 801 and ET-1. However, the reproducibility and reliability obtained by IAsys are better than those obtained by QCM, since the QCM frequency is more sensitive to temperature fluctuations, atmospheric changes and mechanical disturbances. Our work offers a new and effective way to study the interaction of *P. radix* 801 and ET-1 as well as the action mechanism of *P. radix* 801 by the affinity biosensors. There are many advantages of the present methods, such as real time, high sensitivity, high selectivity, high veracity, without usage of label compounds and easy-to-handle. Furthermore, another target that *P. radix* 801 can bind specifically will be clarified by these methods. These methods have tremendous application prospects in studies on the action mechanism of TCM.

## Figures and Tables

**Table 1.** Reproducibility and reliability of IAsys.

**Run**	**Binding mass of ET-1 (ng mm^-2^)**	**Binding mass of *P. radix* 801 (ng mm^-2^)**
1	1.093	1.297
2	1.100	1.306
3	1.096	1.298
4	1.098	1.302
5	1.098	1.302
*x̅*	1.097	1.301
SD	0.002	0.003
RSD(%)	0.18	0.23

(x̅ : mean, SD: standard deviation, RSD: relative standard deviation)

**Table 2.** Reproducibility and reliability of QCM.

**Run**	**Binding mass of ET-1 (ng cm^-2^)**	**Binding mass of *P. radix* 801 (ng mm^-2^)**
1	277.376	1.303
2	280.293	1.317
3	277.262	1.300
4	281.149	1.324
5	278.321	1.310
*x̅*	278.880	1.311
SD	1.571	0.876
RSD(%)	0.56	0.67

(*x̅* : mean, SD: standard deviation, RSD: relative standard deviation)

**Table 3.** Comparison of the IAsys and QCM.

**Item**	**IAsys**	**QCM**
Principle	Resonant mirror optical technology	Mechanical thickness
Response range	Extending ∼300 nm	(sub) Å to µm
Intrinsic sensitivity	200 arc seconds = 1 ng mm^-2^ (CMD-cuvette) 600 arc seconds = 1 ng mm^-2^ (Biotin-cuvette)	1 Hz = 4.4 ng cm^-2^ (in air sensitivity for a 10 MHz crystal)
Environmental effect	Less sensitive	Sensitive
Integration with electrochemical control	Yes	Yes
Response speed	Less fast	Fast
Limitations	Cuvette volume will be limited for some experiments	Liquid phase frequency response is complicated for thin film thickness measurements
Applicability	Concentration determination	Any coating with a viscosity and viscoelasticity contrast with the surrounding medium
	Molecular recognition, binding patterns, co-operativity	Damping is reflective of the viscoelasticity of a coating
	Mapping of multi-molecular interactions, kinetics of association and dissociation	
	epitope mapping	
